# Experimental and Theoretical Insights on the Structural, Electronic, and Magnetic Properties of the Quaternary Selenides EuPrCuSe_3_ and EuNdCuSe_3_

**DOI:** 10.1021/acs.inorgchem.3c04560

**Published:** 2024-05-06

**Authors:** Maxim V. Grigoriev, Anna V. Ruseikina, Alexander A. Garmonov, Ralf J. C. Locke, Filip Sagan, James Hooper, Mariusz P. Mitoraj, Thomas Schleid, Damir A. Safin

**Affiliations:** † Laboratory of Theory and Optimization of Chemical and Technological Processes, University of Tyumen, Volodarskogo Street 6, 625003 Tyumen, Russian Federation; ‡ Institute of Physics and Technology, University of Tyumen, Volodarskogo Street 6, 625003 Tyumen, Russian Federation; § Institute for Inorganic Chemistry, University of Stuttgart, D-70569 Stuttgart, Germany; ∥ Faculty of Chemistry, Jagiellonian University, ul. Gronostajowa 2, 30-387 Krakow, Poland; ⊥ Scientific and Educational and Innovation Center for Chemical and Pharmaceutical Technologies, Ural Federal University Named after the First President of Russia B.N. Yeltsin, Mira Street 19, 620002 Ekaterinburg, Russian Federation; # University of Tyumen, Volodarskogo Street 6, 625003 Tyumen, Russian Federation

## Abstract

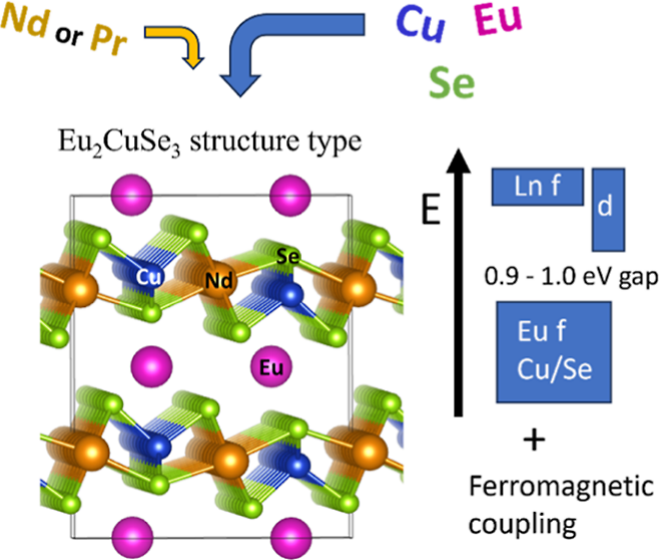

Magnetic semiconductors EuPrCuSe_3_ and EuNdCuSe_3_ were obtained by using the halide flux method. Their crystal structures and magnetic properties were studied and discussed. Optical properties of the obtained selenides were studied by the means of diffuse reflectance spectroscopy, which revealed the values of 1.92/1.97 and 0.90/0.94 eV for the direct and indirect band gaps of Ln = Nd/Pr, respectively. The structural, electronic, and magnetic properties of the obtained compounds were additionally studied with spin-polarized density functional theory calculations, wherein both systems were found to be two new examples of semiconducting quaternary selenides with disperse conduction bands of Nd/Pr 5d character. The modeling showed that various magnetic orderings in the systems have subtle influences on the alignments/overlaps between the Se/Cu, Eu, and Pr/Nd bands, and that the spin-state energetics are very dependent upon the treatment of electron correlation, but a distinguishing feature in the case of ferromagnetic coupling is that the spin density on the Se atoms is maximized. Overall, the calculations are in good agreement with the experimental characterization of ferromagnetism in the bulk crystals, wherein the ferromagnetic transition occurs at temperatures of about 2.5 K for EuPrCuSe_3_ and about 3 K for EuNdCuSe_3_.

## Abstract 2

Filling in the gaps in the series, the crystal structures of EuLnCuSe_3_ with Ln = Pr and Nd are synthesized, characterized, and provided. Both systems were found to adopt the Eu_2_CuSe_3_ structure type and exhibit ferromagnetic coupling with *T*_c_ values of 2–3 K. The indirect band gaps are determined to be ∼0.9 eV, wherein the lowest unoccupied states of the systems are Ln d orbitals modulated by the shortest Ln···Ln contacts.

## 1 Introduction

Over the past 30 years, the search for compounds that combine the properties of semiconductors and ferromagnets has become an important area of materials science. The presence of ferromagnetism in compounds has led to the creation of materials that combine the capabilities of semiconductor quantum structures and ferromagnetic multilayers.^1^ Ferromagnets are one of the most promising materials for magnetic devices and spintronics.^1−6^ Magnetic semiconductors based on lanthanides have already been used in new spintronics applications based on spin-polarized transport,^2^ such as spin transistors and spin filters.^4^ Rare-earth (RE) element selenides are known as frustrated magnets and spin glasses.^5^ Understanding and controlling frustrated magnetism could lead to the creation of new materials with unusual properties and improvements in existing information storage and processing technologies. Density functional theory (DFT) studies of RE element selenides revealed two band gap channels (Eg_1_ and Eg_2_) for the main and minor spin bands, which is of great importance for applications in spintronics.^6^

Similar magnetic properties can be revealed in more complex systems based on RE element chalcogenides.^7−19^ For quaternary europium chalcogenides EuLnCuCh_3_ (Ch = S, Se), it has been suggested that if the magnetic moment of the Ln^3+^ ion is significantly less than the magnetic moment of the Eu^2+^ ion, the compound will be ferromagnetic, and if the magnetic moment of the Ln^3+^ ion is equal to or greater than that of the ion Eu^2+^, the compound exhibits ferrimagnetic properties.^20−24^ Chalcogenides EuLnCuCh_3_ (Ch = S, Se, Te), containing nonmagnetic ions Ln^3+^ = La^3+^, Y^3+^, Lu^3+^ and Sc^3+^, exhibit exclusively ferromagnetic ordering of the Eu^2+^ moments with the ground state 8S_7/2_ at 2.4–6.4 K.^12,13,20,22,23^ However, chalcogenides with magnetic ions Ln^3+^ = Ce^3+^–Sm^3+^ and Gd^3+^–Yb^3+^ exhibit both ferro- and ferrimagnetic behavior at 1.7–4.3 K and 4.5–6.2 K, respectively.^12,13,20−22,25^ In the L-^12^ and N-type ferrimagnets, according to the Néel classification,^26^ the effect of negative magnetization was discovered.^21^ In EuPrCuSe_3_ and EuNdCuSe_3_, the effective magnetic moments of Pr^3+^ (3.58 μB) and Nd^3+^ (3.62 μB)^20^ are significantly lower than that of the Eu^2+^ ion (7.94 μB), which suggests the presence of a ferromagnetic transition in these compounds.

To the best of our knowledge, the magnetic properties of EuPrCuSe_3_ and EuNdCuSe_3_ have not yet been studied. For EuPrCuSe_3_ and EuNdCuSe_3_, crystallization in two space groups *Pnma* and *Cmcm* with structure types BaLaCuS_3_ and KZrCuSe_3_, respectively, was predicted using ab initio calculations.^27^ EuRECuSe_3_, except EuPrCuSe_3_ and EuNdCuSe_3_, were also prepared using the synthetic reductive selenidation approach, although they contained some impurities (<5%).^21,24^ Recently, a polycrystalline sample of EuNdCuSe_3_ was synthesized.^19^ However, the resulting product was contaminated with NdCuSeO, EuNd_2_Se_4_, and EuNdSe_2_ (12.6%). With all of this in mind, in this work we have focused on the synthesis of pure EuPrCuSe_3_ and EuNdCuSe_3_ as well as studying their crystal structures and magnetic properties. Additionally, computational studies were preformed to shed light on the electronic structure and the related magnetic properties. Understanding the electronic and magnetic properties of EuLnCuSe_3_ (Ln = Pr, Nd) can provide insight into its application in magnetic and spintronic materials.

## 2 Experimental Section

### 2.1 Materials

Eu (99.3%), Pr (99.9%), Nd (99.9%), Se (99.9%), and CsI (99.9%) were purchased from ChemPur (Karlsruhe, Germany). Cu (99.999%) was obtained from Aldrich (Milwaukee, USA).

### 2.2 Diffuse Reflectance Spectroscopy

The diffuse reflectance spectra were recorded using a UV-2600 spectrophotometer (Shimadzu OJSC, Tokyo, Japan) equipped with an ISR-2600Plus attachment with the photomultiplier PMT of the R-928 type and InGaAs detectors. BaSO_4_ (99.8%) was used as a reference.

### 2.3 X-ray Diffraction Analysis

The single-crystal X-ray diffraction data for EuPrCuSe_3_ and EuNdCuSe_3_ of 0.05 × 0.05 × 0.45 mm^3^ dimensions (Figure S1 in the Supporting Information) were collected at 293(2) K with a Bruker–Nonius κ-CCD diffractometer (Mo Kα radiation, graphite monochromator) equipped with a CCD detector. The collected intensity data and the numerical correction of the absorption for the measured crystals were processed using the DENZO^28^ and HABITUS^29^ programs, respectively. The crystal structures were solved and refined using the SHELX-2013 software package.^30,31^

CCDC 2207233 and 2207234 contain supplementary crystallographic data. These data can be obtained free of charge via https://www.ccdc.cam.ac.uk/structures or from the Cambridge Crystallographic Data Centre, 12 Union Road, Cambridge CB2 1EZ, UK; fax: (+44)-1223–336–033; or e-mail: deposit@ccdc.cam.ac.uk.

### 2.4 Powder X-ray Diffraction Analysis

The powder X-ray diffraction data (Figure S2 in the Supporting Information) were collected at room temperature with a DRON 7 (Innovation Center Bourevestnik, Saint-Petersburg, Russian Federation) powder diffractometer (Cu Kα radiation, graphite monochromator). The step size of 2θ was 0.02° and the counting time was 10 s/step. Rietveld refinement was used to refine the structures.

### 2.5 Electron-Beam Microprobe Analysis

The SEM images were acquired by using an electron-beam X-ray microprobe (SX-100, Cameca, Gennevilliers, France). The EDX spectra for several examples roughly confirmed the 1:1:1:3 stoichiometries of the discussed compounds.

### 2.6 Magnetic Measuremenets

The temperature-dependent (2–300 K) magnetic susceptibilities of EuPrCuSe_3_ and EuNdCuSe_3_ were studied using a Quantum Design MPMS3 SQUID magnetometer in a 500 Oe magnetic field. The measurements were performed in the zero-field-cooled (ZFC) and nonzero-field-cooled (FC) modes. The field-dependent magnetic susceptibilities of EuPrCuSe_3_ and EuNdCuSe_3_ were studied at 2 and 300 K on a vibrating sample magnetometer within the same Quantum Design MPMS3 SQUID magnetometer.

### 2.7 DFT Computations

DFT was used to model the periodic (EuPrCuSe_3_)_4_ and (EuNdCuSe_3_)_4_ systems, primarily within the scope of the PBE + D3 + *U*(32−34) density functional methodology as it is programed within the VASP computational program (version 6.3.1^35^), and supplemented with the SCAN^36^ and HSE06^37^ density functionals as well as the modified Becke–Johnson (MBJ) potential.^38^ Two different sets of computational parameters were employed: one set for screening various potential magnetic states of the systems using the cell parameters that were obtained at 298 K and the second set for examining how the electronic structure is affected by changes in methodology. For the set of calculations whose intent is to screen magnetic states, the plane-wave basis set cutoff was set to 350 eV, the Eu/Nd 5s/5p/6s/5d/4f, Se 4s/4p, and Cu 4s/3d valence electrons were treated explicitly and used in conjunction with PAW potentials^39^ that were supplied with the standard VASP package (version 5.4), the Brillouin zone was sampled using a 2 × 4 × 2 *k*-point mesh, and the symmetry that was detected by VASP (concerning the initial geometry and assignment of magnetic configuration) was preserved in all calculations. The second set of calculations on the ferromagnetic (F) and ferrimagnetic (FE) states used a basis set cutoff of up to 400 eV and sampled *k*-point meshes of up to 4 × 9 × 3 in size for selected systems. Complementary results that describe how the key computational conclusions in this work are affected by the choice of PAW potential are given in Supporting Information. The VESTA program^40^ was used to generate the electron density plots.

### 2.8 Synthesis

No uncommon hazards are noted. Crystals of EuPrCuSe_3_ and EuNdCuSe_3_ were obtained from a stoichiometric ratio of the elemental europium, copper, praseodymium, or neodymium and selenium in the presence of CsI as a flux. The silica ampules were evacuated to a pressure of 2 × 10^–3^ mbar and sealed. They were then heated in a muffle furnace from room temperature to 1120 K for 30 h and kept at this temperature for 96 h, and after that cooled to 570 K at a rate of 4 K/h, then to room temperature within 3 h. The reaction product was purified from flux residues with demineralized water. The resulting dark red needle-like crystals were suitable for a single-crystal X-ray diffraction analysis.

## 3 Results and Discussion

The heterometallic quaternary selenides EuPrCuSe_3_ and EuNdCuSe_3_ were readily obtained from a stoichiometric mixture of the parent elements in the presence of CsI as a flux upon heating at 1070 K for 6 days. This synthetic approach allowed production of crystals suitable for a single-crystal X-ray diffraction analysis without further purification of the final product.

According to the single-crystal X-ray diffraction analysis data, the title selenides are isostructural and crystallize in the orthorhombic space group *Pnma* with the Eu_2_CuS_3_ structure type (Table 1). The asymmetric unit cell contains one europium, one copper, one praseodymium or neodymium, and three selenium ions (Table S1 in the Supporting Information).

**Table 1:** Experimental Details for the Structures of EuPrCuSe_3_ and EuNdCuSe_3_

	EuPrCuSe_3_	EuNdCuSe_3_
molecular weight	593.29	596.62
space group	*Pnma*	
structural type	Eu_2_CuSe_3_	
*a* (Å)	10.9151(1)	10.8696(1)
b (Å)	4.1400(5)	4.1310(4)
c (Å)	13.3572(7)	13.3659(6)
V (Å3)	603.59(8)	600.16(6)
*Z*	4	4
ρ (g cm^–3^)	6.529	6.603
μ (mm^–1^)	39.636	40.396
collected reflections	12,684	11,772
unique reflections	785	780
*R*_int_	0.1108	0.0798
*R*_1_ (all)	0.0504	0.0367
*wR*_2_ (all)	0.0659	0.0610
*S*	1.018	1.020

**Figure 1:**
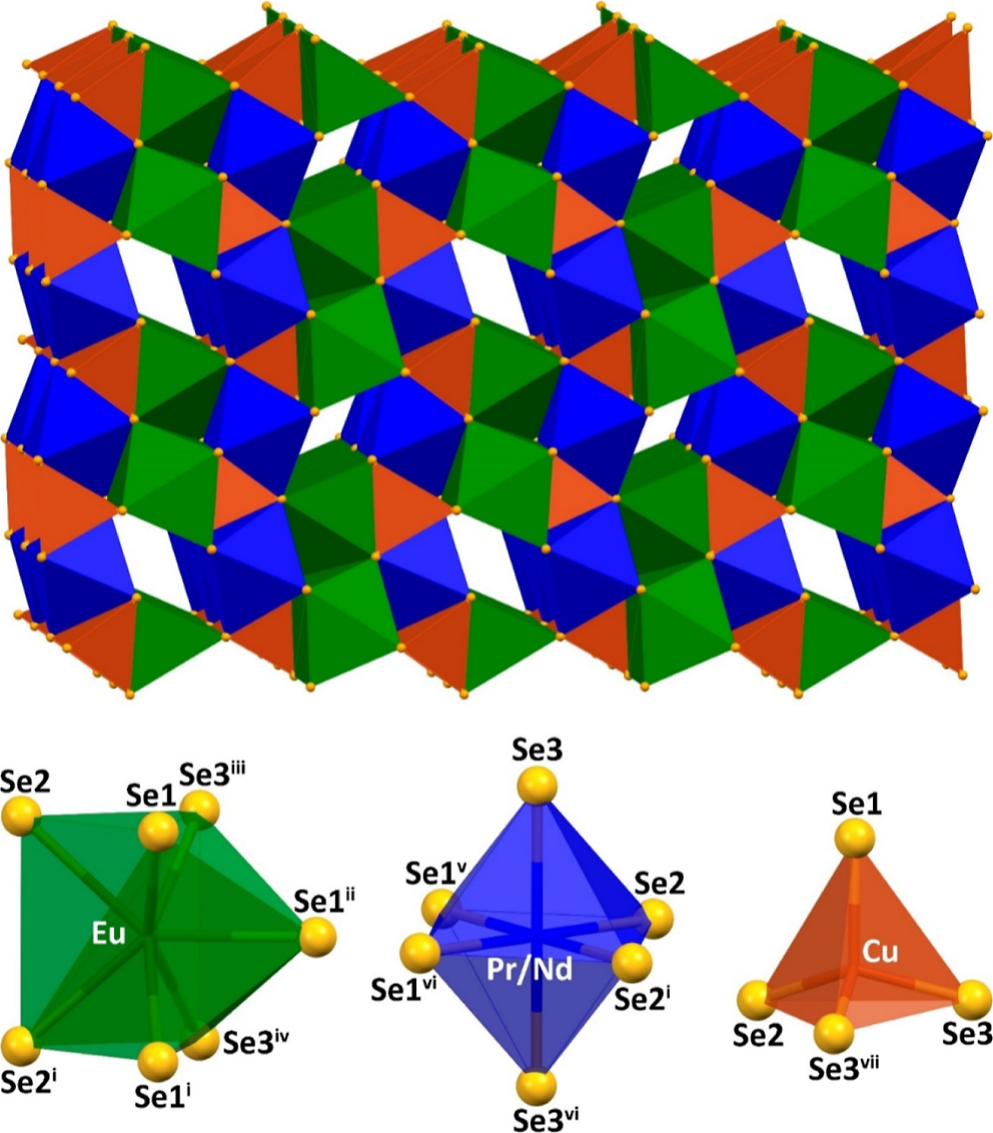
View of the crystal structures of EuPrCuSe_3_ and EuNdCuSe_3_ along the *b* axis, together with the coordination polyhedra formed by the metal ions. Color code: green polyhedron = EuSe_7_, blue polyhedron = Pr/NdSe_6_, and burnt orange polyhedron = CuSe_4_. Symmetry codes: (i) *x*, −1 + *y*, *z*; (ii) −*x*, −1/2 + *y*, 2 – *z*; (iii) 1/2 – *x*, −*y*, 1/2 + *z*; (iv) 1/2 – *x*, −1 – *y*, 1/2 + *z*; (v) 1/2 + *x*, 1/2 – *y*, 3/2 – *z*; (vi) 1/2 + *x*, −1/2 – *y*, 3/2 – *z*; (vii) *x*, 1 + *y*, *z*.

A 3D crystal structure of the discussed selenides is constructed from the EuSe_7_ capped trigonal prisms, Pr/NdSe_6_ distorted octahedra as well as CuSe_4_ tetrahedra (Figure 1). The Pr/NdSe_7_ capped trigonal prisms form 2D layers within the *ab* plane, further strengthened by 1D polymeric chains (CuSe_4_)_*n*_ (Figure 1). These layers are separated by 1D dimeric ribbons, formed by the EuSe_7_ capped trigonal prisms, and 1D free channels along the *b* axis (Figure 1).

In the discussed structures, the Eu–Se bond lengths within the trigonal prism are 3.1040(3)–3.1808(4) Å, while the seventh Eu–Se bond is remarkably longer and of 3.2935(4)–3.3063(3) Å (Table 2). The Pr/Nd–Se and Cu–Se bond lengths in both structures are similar, and of 2.8976(3)–2.9591(4) Å and 2.4703(3)–2.5244(3) Å, respectively (Table 2). The Se–Pr/Nd–Se bond angles within the Pr/NdSe_6_ octahedra are either close to 90° (86.50–95.65°) or close to 180° (172.54–176.56°) (Table S2 in the Supporting Information). Five Se–Cu–Se bond angles within the CuSe_4_ tetrahedra in both structures are close to the ideal tetrahedral angle and vary from 110.13 to 111.58°, while the sixth Se–Cu–Se bond angle is somewhat smaller and of 102.79–102.87° (Table S2 in the Supporting Information). Finally, the Se–Eu–Se bond angles within the EuSe_7_ capped trigonal prisms vary in a broad range from 72.08 to 151.54° (Table S2 in the Supporting Information).

**Table 2:** Bond Lengths (Å) in the Crystal Structures of EuPrCuSe_3_ and EuNdCuSe_3_^a^

EuPrCuSe_3_	Eu–Se1	3.1590(4)	Pr–Se1^v^	2.9591(4)	Cu–Se1	2.4703(3)
	Eu–Se1^i^	3.1590(4)	Pr–Se1^vi^	2.9591(4)	Cu–Se2	2.4562(3)
	Eu–Se1^ii^	3.2935(4)	Pr–Se2	2.9068(4)	Cu–Se3	2.5244(3)
	Eu–Se2	3.1808(4)	Pr–Se2^i^	2.9068(4)	Cu–Se3^vii^	2.5244(3)
	Eu–Se2^i^	3.1808(4)	Pr–Se3	2.9354(4)		
	Eu–Se_3_^iii^	3.1075(4)	Pr–Se3^vi^	2.9362(4)		
	Eu–Se3^iv^	3.1075(4)				
EuNdCuSe_3_	Eu–Se_1_	3.1596(3)	Nd–Se1^v^	2.9473(3)	Cu–Se1	2.4706(2)
	Eu–Se1^i^	3.1596(3)	Nd–Se1^vi^	2.9473(3)	Cu–Se2	2.4537(2)
	Eu–Se1^ii^	3.3063(3)	Nd–Se2	2.8976(3)	Cu–Se3	2.5195(2)
	Eu–Se2	3.1785(3)	Nd–Se2^i^	2.8976(3)	Cu–Se3^vii^	2.5195(2)
	Eu–Se2^i^	3.1785(3)	Nd–Se3	2.9248(3)		
	Eu–Se3^iii^	3.1040(3)	Nd–Se3^vi^	2.9248(3)		
	Eu–Se3^iv^	3.1040(3)				

a For symmetry codes, see Figure 1.

**Figure 2:**
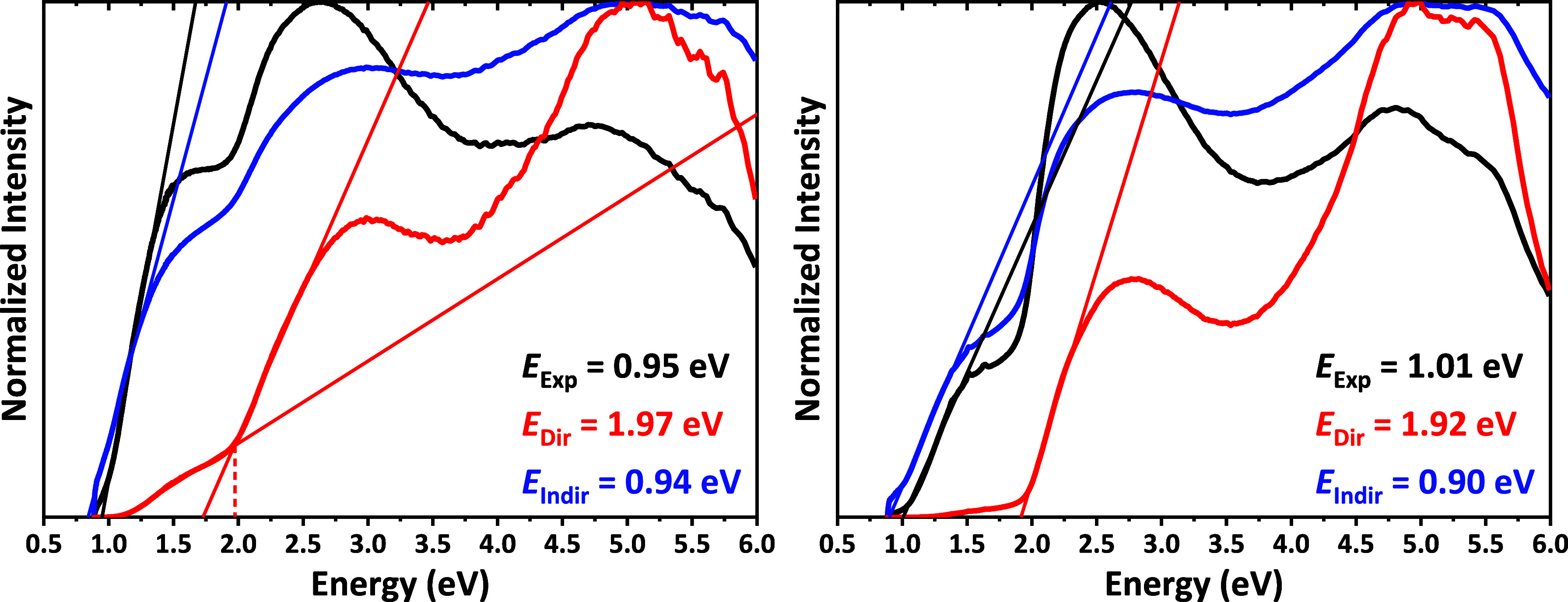
Normalized Kubelka–Munk (black) and (Kubelka–Munk × energy)^a^ (*a* = 2, red; 1/2, blue) spectra of EuPrCuSe_3_ (left) and EuNdCuSe_3_ (right).

The corresponding band gaps of the two selenides obtained herein were revealed from the normalized Kubelka–Munk spectra shown in Figure 2. From these plots, it was established that the direct and indirect band gaps are very similar for both selenides, wherein the direct gaps were in the range of 1.92–1.97 eV and the indirect gaps in the range of 0.90–0.94 eV.

**Figure 3:**
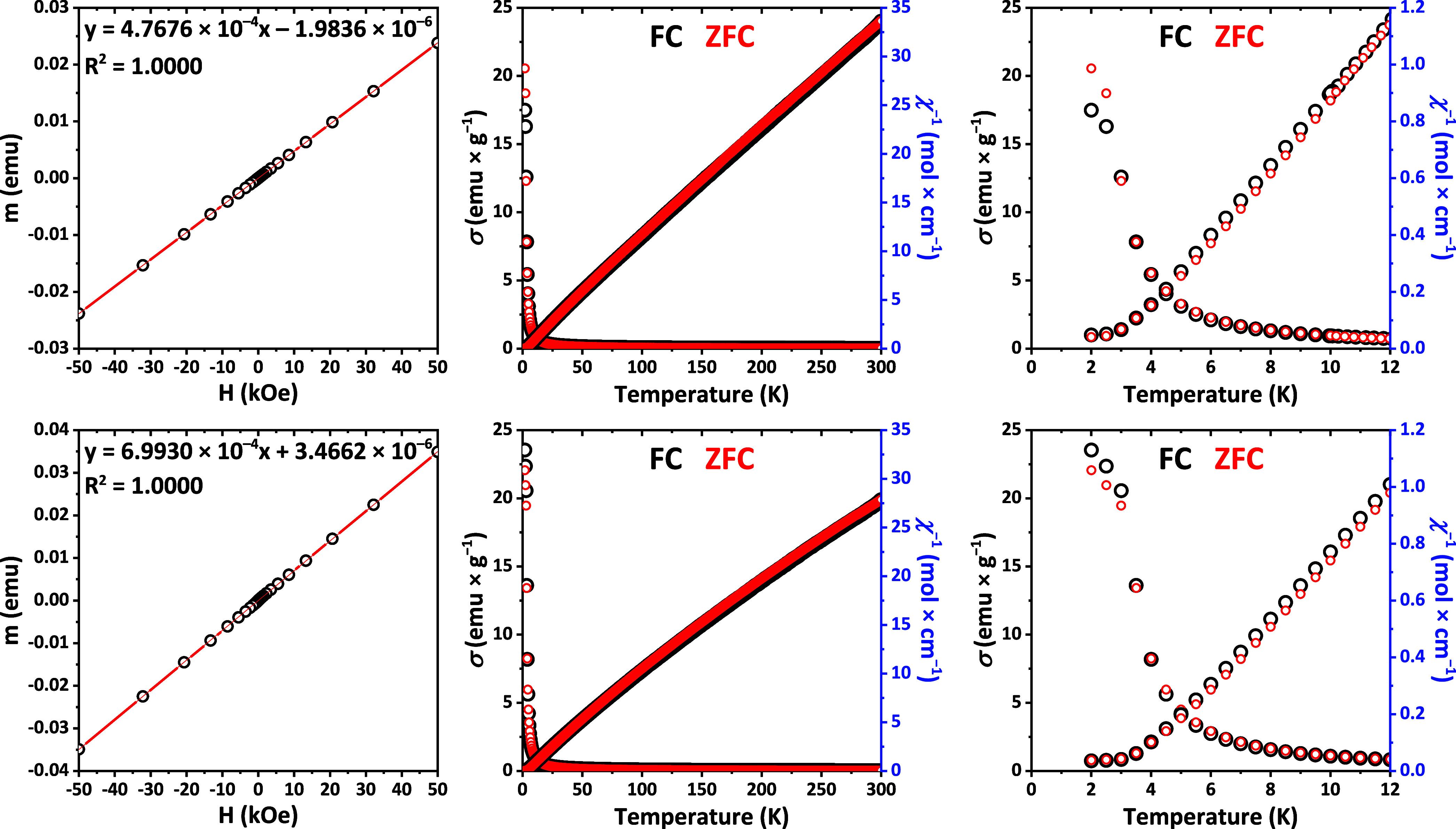
Field-dependent magnetic moments at 300 K (left), and temperature-dependent specific magnetization and reciprocal magnetic susceptibility (middle and right) of EuPrCuSe_3_ (top) and EuNdCuSe_3_ (bottom) at 500 Oe. The temperature-dependent measurements were performed in the ZFC and FC modes.

The temperature-dependent magnetization of the EuPrCuSe_3_ and EuNdCuSe_3_ samples is very similar, and the temperature-dependent reciprocal magnetic susceptibilities are well described by the Curie–Weiss law and are the same in both the ZFC and FC modes (Figure 3). As such, the μ, *C* and θ values were calculated for both compounds at 60–300 K (Table 3). The values of the Weiss constants θ for both compounds are very close to zero; thus, there is almost no coordination of magnetic moments in this temperature range, which is typical for ideal paramagnets. However, the values of the Curie constant (*C*_60–300 K_) and the effective magnetic moments (μ_60–300 K_) are lower than those for the noninteracting Pr^3+^ and Nd^3+^ magnetic centers, though the deviation is not very large (Table 3). Notably, both temperature dependences exhibit a clearly distinguished feature at temperatures below 4 K, where a change in the slope of the curve and a discrepancy in the data for the ZFC and FC modes are observed (Figure 3). Thus, most likely, these compounds undergo a transition to a ferromagnetic state at about 2.5 K for EuPrCuSe_3_ and at about 3 K for EuNdCuSe_3_. This conclusion is supported by a comparison with the data recently obtained for sulfides EuPrCuS_3_ and EuNdCuS_3_, for which a ferromagnetic transition was observed at 2.1 and 3.1 K, respectively.^20^ The field-dependent magnetic moment of both EuPrCuSe_3_ and EuNdCuSe_3_ at 300 K is linear, which is characteristic for a paramagnetic compound (Figure 3). The Curie constants and effective magnetic moments calculated from these dependences are very close to those revealed from the temperature dependences (Table 3), which is consistent with the Weiss temperature values being close to zero.

**Table 3:** Magnetic Characteristics for EuPrCuCh_3_ and EuNdCuCh_3_ (Ch = S, Se)

	EuPrCuS_3_^20^	EuPrCuSe_3_	EuNdCuS_3_^20^	EuNdCuSe_3_
space group	*Pnma*			
structural type	Ba_2_MnS_3_	Eu_2_CuS_3_	Ba_2_MnS_3_	Eu_2_CuS_3_
calculated μ (μ_B_)	8.706		8.723	
experimental μ_300 K_ (μ_B_)		8.28		8.46
experimental μ_60–300_ K(μ_B_)	8.68	8.23	8.73	8.46
calculated *C* (K cm^3^ mol^–1^)	9.479		9.515	
experimental *C*_300 K_ (K cm^3^ mol^–1^)		8.57		8.96
experimental *C*_60–300 K_ (K cm^3^ mol^–1^)	9.42 (20–50 K)	8.47	9.52 (20–50 K)	8.85
experimental θ (K)	0.5	0.5	3.2	0.8
experimental *T*_c_ (K)	2.1	2.5	3.1	3.0
arrangement type	ferromagnetic		ferromagnetic	

**Figure 4:**
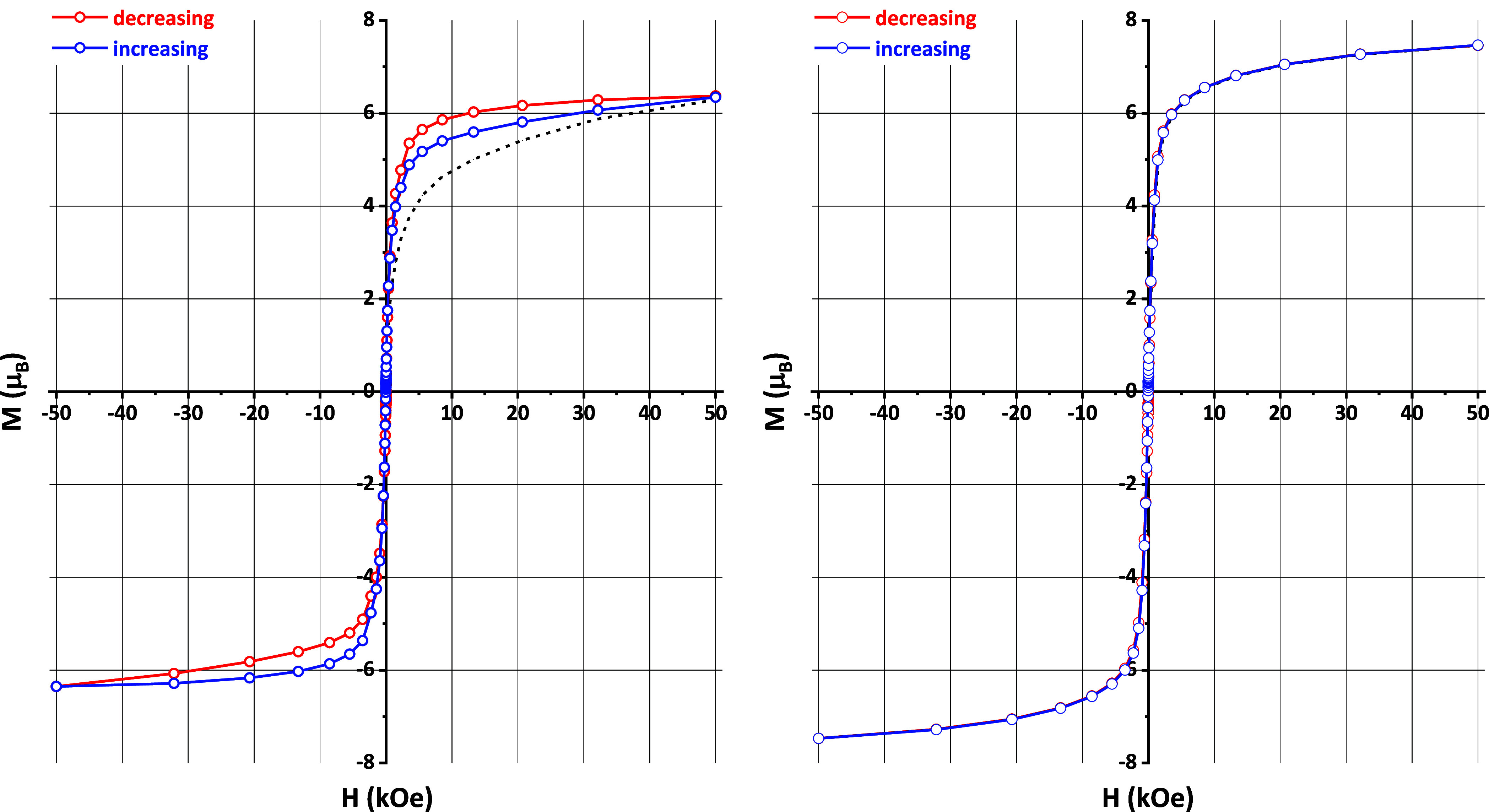
Magnetization curves of EuPrCuSe_3_ (left) and EuNdCuSe_3_ (right) at 2 K.

The field-dependent magnetization curves measured at 2 K are characteristic of a “soft” ferromagnet for both selenides (Figure 4). Saturation starts at about 5 kOe. For EuPrCuSe_3_ and EuNdCuSe_3_, the magnetic moment, calculated per one formula unit, is about 6.5 and 7.5 μB, respectively (Figure 4). However, the total magnetic moment for two types of magnetic cations is gS(Eu^2+^) + g_J_J(Pr^3+^) = 7 + 16/5 = 10.20 μ_B_ and gS(Eu^2+^) + g_J_J(Nd^3+^) = 7 + 36/11 ≈ 10.27 μB for EuPrCuSe_3_ and EuNdCuSe_3_, respectively. Such a significant difference is explained by the fact that the measurements were carried out at the temperature near the Curie points, where the magnetization saturation has a sharp drop. The coercive force in both compounds does not exceed the measurement error of about 20 Oe.

**Figure 5:**
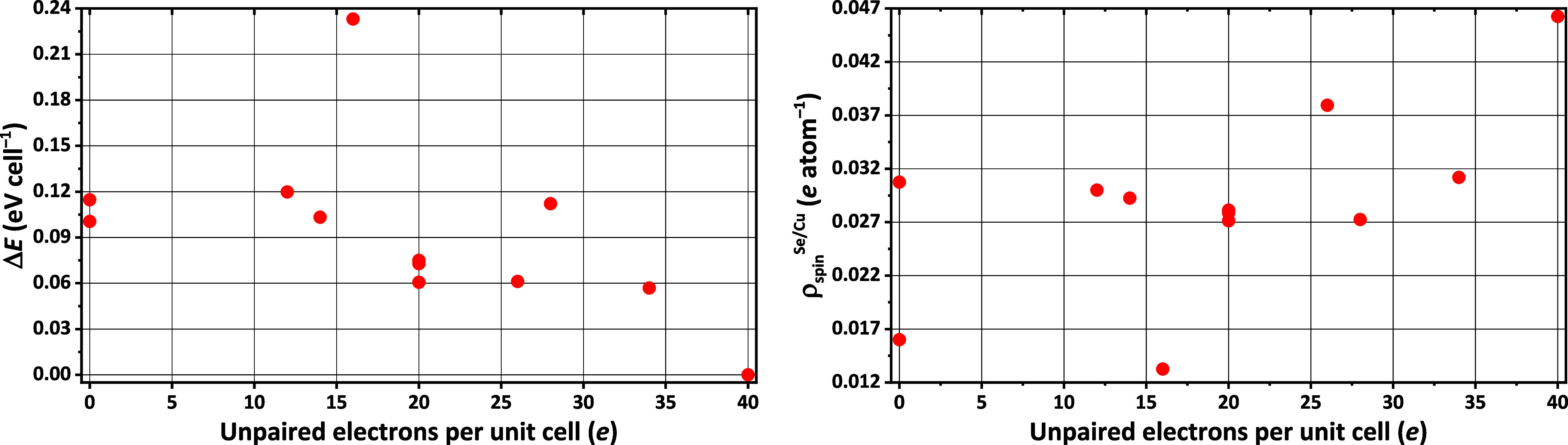
(Left) A plot of the energy differences between distinct (single-determinant) electron configurations of (EuNdCuSe_3_)_4_ as computed at the DFT/PBE + D3 level of theory. (Right) A plot of the average number of unpaired electrons per atom attributed to the Se/Cu atoms at the optimized geometry of each electron configuration. ρ_spin_^Se/Cu^ refers to the portion of the spin density that is attributed to atom Se or Cu and the data was generated from the default population analysis scheme that is printed in the VASP output. In both plots, the F state appears at *x* = 40 *e* and the FE state appears at *x* = 16 *e*.

DFT calculations were first used to look for electronic structure features that relate to the ferromagnetic ordering that is seen in experiment. To this aim, we applied the DFT/PBE + D3 method to explore different potential magnetic states of EuNdCuSe_3_. The PBE + D3 functional was chosen because the PBE functional is a well-established method that is routinely used in solid-state research studies that characterize lattice parameters and phase diagrams in related systems.^6,27^ This type of DFT requires that the electronic orbitals, which are used to expand the density and energy expressions, are assigned formal occupation numbers within a “single-determinant” formalism. With regard to modeling magnetism, this means that the relevant occupied and unoccupied spin orbitals that contribute to the magnetism must be definitively assigned to particular atomic centers. This sets up something of a coloring problem, wherein each atom-centered set of unpaired 4f spin orbitals on Eu and Pr/Nd could be assigned as either alpha or beta spin. To confirm that the PBE + D3 level of theory ably predicts that the ferromagnetic (F) configuration (i.e., the state with 40 unpaired electrons in the unit cell at the far right-hand side of the *x*-axis) is the most stable (Figure 5), different possible assignments of alpha and beta spin orbitals were assigned within the unit cell of (EuNdCuSe_3_)_4_ and their total energies were computed and compared (the unit cell parameters were kept fixed at the experimental 298 K values in order not to bias the results toward any single optimized state).

The stability of the F state agrees with the experimental characterization of ferromagnetism in the bulk crystals at a low temperature. The least stable antiferromagnetic state is a ferrimagnetic (FE) one wherein all of the unpaired Eu and Nd electrons are the same spin among themselves but differ in spin between them, giving rise to an excess of 16 unpaired electrons per unit cell (Figure 5). It was noted that F and FE correspond with the states that have the most and least spin densities, respectively, within the Se/Cu sublattice (Figure 5). The FE state will therefore be used here as a means of exploring the nature of the ferromagnetic coupling in two ways: first, by defining an energy difference between F and FE that helps to quantify the magnetic coupling between cations and, second, by comparing differences in the states’ electron densities.

**Figure 6:**
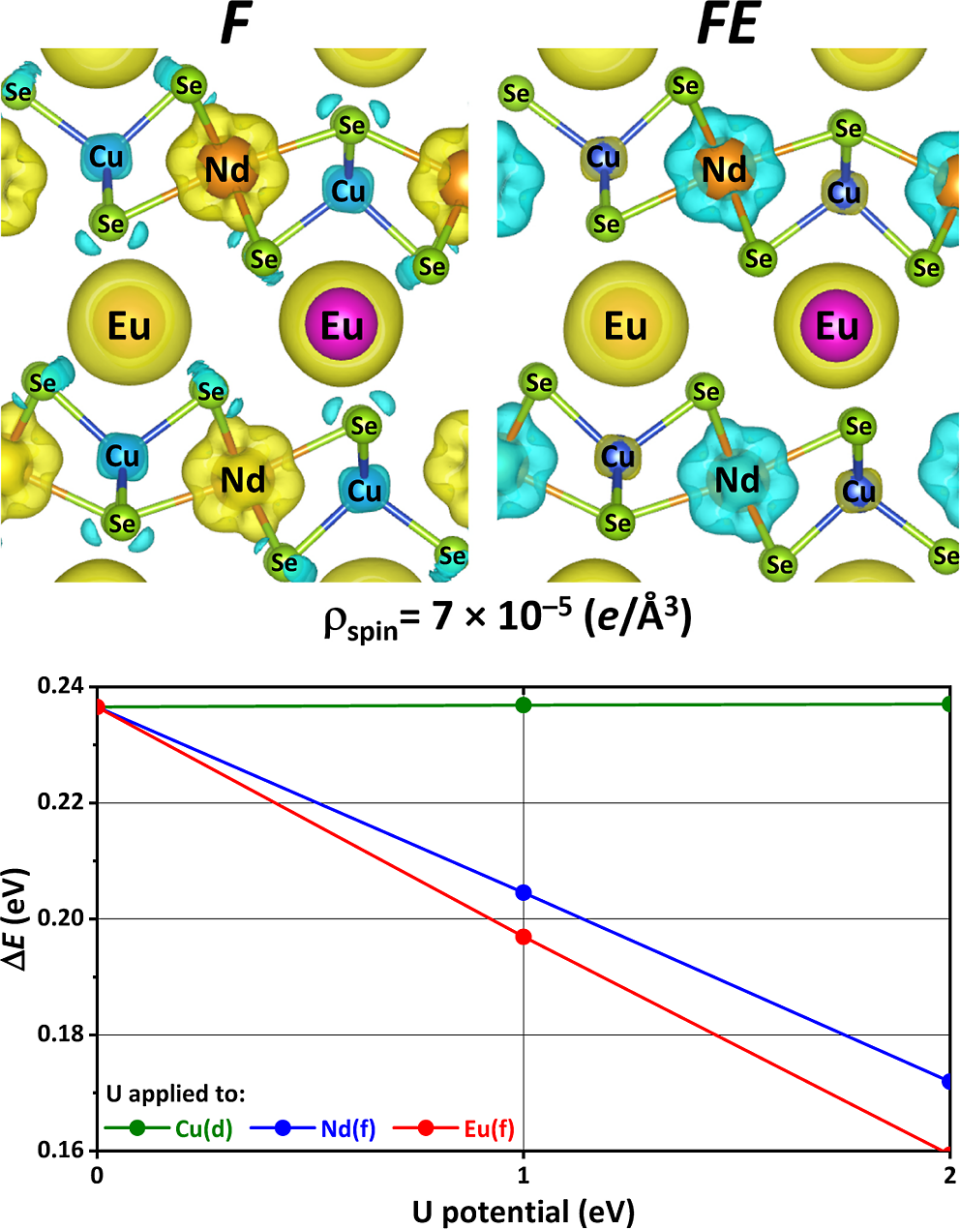
(Top) Isosurface plots of the spin densities (ρ_spin_) for states F and FE of (EuNdCuSe_3_)_4_ (ρ_spin_ = ρ_α_ – ρ_β_, where ρ_α_ is the electron density generated by the α-spin orbitals and ρ_β_ is the electron density generated by the β-spin orbitals). The yellow isosurface corresponds to the +0.00007 e/Å^3^ isosurface (i.e., an excess of α-electron density) and the blue isosurface corresponds to the −0.00007 e/Å^3^ isosurface (i.e., an excess of β-electron density). (Bottom) A plot of how the energy difference between F and FE changes as a Hubbard potential (*U*) is introduced into the selected atomic orbitals.

The spin densities (ρ_spin_) for the F and FE states of (EuNdCuSe_3_)_4_ at the PBE + D3 level of theory clearly show that the Eu and Nd orbitals are the largest overall contributors, but the most visible differences between them indeed concern the spin density within the Se/Cu sublattice; contributions from the Se and Cu atoms are clearly visible in F but not in FE (Figure 6). This visually confirms the trend noted above about the relation between the spin density on the Se/Cu sublattice and the apparent strength of the magnetic coupling between cations. It further shows that the PBE + D3 method is able to at least qualitatively describe that the Se atoms are contributing to the magnetism via direct exchange and/or superexchange mechanisms. The ferromagnetic coupling within a metallic RE framework suggests that there are likely multiple coupling mechanisms involving filled, half-filled, and unfilled atomic 4f orbitals, but we refrain from further characterization of them in this work as it is well-known that such analyses require accurate treatments of electron correlation of both dynamic and static types, as implied by the closeness of the energies in the states (Figure 5) and of spin–orbit relativistic effects.

The stability of the F state was further confirmed in a 1 × 2 × 1 supercell of the (EuNdCuSe_3_)_4_ crystal structure, as this choice of cell allows us to explore antiferromagnetic coupling of the shortest Eu···Eu and Nd···Nd (Table S3 in the Supporting Information). As could be expected, the energy difference between the F and FE states of (EuNdCuSe_3_)_4_ is affected by the use of local atom-centered Hubbard potentials on selected sets of atomic orbitals within the popular DFT + *U* scheme (Figure 6). An increase in the Hubbard parameter (*U*) is often used as a tool to tune orbital interactions between the targeted orbitals and the surrounding environment, and it is seen that the magnetic coupling, as measured by the difference in energy between F and FE, is weakened by applying progressively stronger Hubbard potentials to the Eu/Nd 4f states (Figure 6). This weaker coupling relates better with the low *T*_c_ from experiment and with what we expect of higher-level DFT methodologies, and it corroborates that the magnetic coupling is directly linked to the ability of the Eu and Nd 4f orbitals to interact with the crystal environment. The use of the DFT + *U* method on the Cu d orbitals exhibited little to no effect on the difference in energy between the F and FE states.

The use of DFT + *U* also opens up a band gap that relates better with experiment. Table 4 shows that the computed band gaps at different levels of theory wherein the DFT + *U* method was used in all cases with *U* = 4.0 eV potentials assigned to the Nd/Pr/Eu f states and *U* = 2.0 eV potentials assigned to the Cu d states; here the band gap is defined as the difference between the conduction band minimum (CBM) and valence band maximum (VBM), and these settings of the *U* parameters were selected as a simple assignment scheme that causes all three DFT methods we are using with it (i.e. PBE, SCAN, and MBJ) to reproduce agreeable band gaps with experiment (note that the band gaps are <0.2 eV in the absence of the +*U* correction when using these GGA and metaGGA functionals, see the expanded results in the Supporting Information). This implementation of the PBE + D3 + *U* method, for example, predicts a band gap of ∼0.8 eV (which is already close to the indirect gap that was deduced from experiment), and it maintains that F exhibits a higher spin density on the Cu/Se sublattice than FE, although the two states, F and FE, become very close in energy. MetaGGA functionals, especially the MBJ + *U* functional, have become more popular in modeling other types of quaternary selenides in the recent literature.^41,42^ As such, we also show the computed properties of (EuNdCuSe_3_)_4_ with the MBJ + *U* and SCAN + *U* methodologies (Table 4). These metaGGA functionals predict a slightly higher band gap than PBE, using the same set of Hubbard potentials, but overall, they exhibit similar tendencies with respect to the spin-state energetics and spin densities that were discussed with PBE + D3 + *U*. The hybrid HSE06 functional also reproduces such behaviors but exhibits a higher band gap than experiment. Spin–orbit coupling was evaluated with the PBE + D3 + *U*, SCAN + *U* and HSE06 methods, and it consistently lowered the predicted band gaps by ∼0.1 eV. Further details and results with other choices of methodology are given in the Supporting Information (Table S4 and Figures S3 and S4). Altogether, we highlight here that this choice of the SCAN + *U* method with spin–orbit coupling corrections gives a band gap of 0.96 eV, which agrees fairly well with experiment. The PBE + D3 + *U* and SCAN + *U* methods were further used to model (EuPrCuSe_3_)_4_, and the results were found to agree both qualitatively and quantitatively with the results of (EuNdCuSe_3_)_4_ (Table 4 and Figure S5 of the Supporting Information).

**Table 4:** Computed Spin-State Energetics, Band Gaps and Spin-Densities of (EuNdCuSe_3_)_4_ and (EuPrCuSe_3_)_4_ with Different Types of DFT Methods^a^

method	*E*_F_ – *E*_FE_ (eV)	[*E*_CBM_ – *E*_VBM_]_F_ (eV)	|ρ_spin_^Se/Cu^|_F_ (e/atom)	|ρ_spin_^Se/Cu^|_FE_ (*e*/atom)
(EuNdCuSe_3_)_4_				
PBE + D3 + *U*^b^	–0.06	0.80	0.032	0.004
PBE + D3 + *U* w/soc^b^	–0.06	0.73	0.032	0.004
HSE06^c^	–0.09	1.37	0.024	0.003
MBJ + *U*^b^,^c^		1.14	0.017	0.008
SCAN + *U*^b^,^c^	–0.01	1.02	0.041	0.003
SCAN + *U* w/soc^b^,^c^	–0.04	0.96	0.041	0.003
(EuPrCuSe_3_)_4_				
PBE + D3 + *U*^b^	–0.02	0.84	0.028	0.003
SCAN + *U*^b^,^c^	–0.01	1.08	0.039	0.004

a *E*_F_ – *E*_FE_ shows the difference between the total DFT energy of the F and FE states, [*E*_CBM_ – *E*_VBM_]_F_ shows the computed band gap of the F state, and |ρ_spin_^Se/Cu^|_F_ and |ρ_spin_^Se/Cu^|_FE_ are measures of the spin density on the Se/Cu sublattice in the F and FE states. The “w/soc” tag indicates that spin–orbit coupling was included in the calculation.

b DFT + *U* indicates that Hubbard-like potentials were used (within the framework of the GGA + *U* method) on Nd/Pr/Eu f states with *U* = 4.0 eV and on Cu d states with *U* = 2.0 eV.

c These functionals were evaluated at the optimized PBE + D3 + *U* geometry.

The DFT results overall suggest that the F model is only marginally, if at all, preferred over the FE model in these systems (relatedly, we note that SCF convergence was very slow and for the most part only moderate energy convergences, of at least 10^–4^, were reached). Furthermore, the differences in F/FE spin-state energetics are seen to overlap with its sensitivity to the choice of functional, choice of Hubbard potential parameters, and choice of PAW potential (Table S4 in the Supporting Information). This serves as a reminder that the electron correlation effects in these systems are complex. Nonetheless, the distinguishing features of the spin density and of the valence/conduction bands seem to agree both qualitatively and quantitatively among the DFT methods (the standout being the MBJ + *U* method; see the discussion of Figure S3 in the Supporting Information).

**Figure 7:**
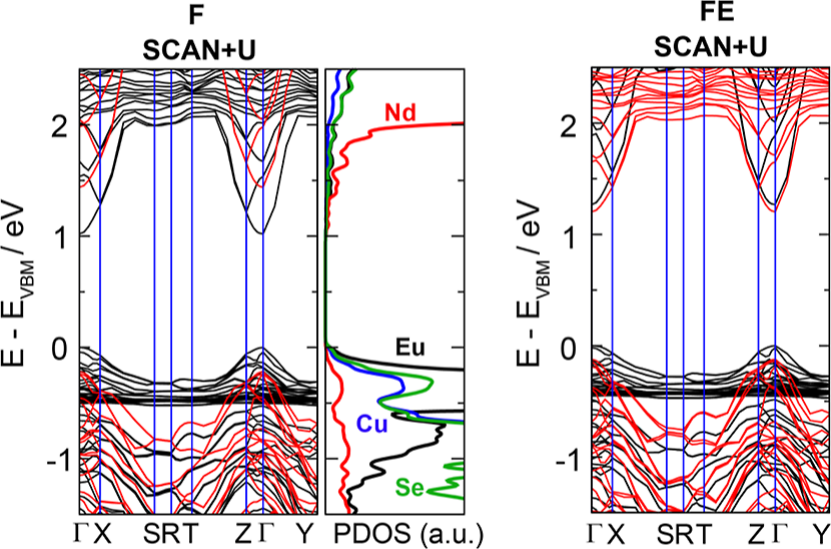
Computed electronic band structure for (left) the F state and (right) the FE state of (EuNdCuSe_3_)_4_ with the experimental 298 K cell parameters at the SCAN + *U*/PBE + D3 + *U* level of theory. The VBM is set to zero, and the two sets of spin orbitals are (separately) colored black and red. The site-projected (partial) density of states is shown alongside the band structure plot of the F state.

The computed electronic band structures of the F and FE states of (EuNdCuSe_3_)_4_ at the SCAN + *U* level of theory are shown in Figure 7. They confirm its semiconducting nature, wherein the Eu 4f bands and Nd 4f bands lie in the ranges (−0.5, 0.0) and (>2.0 eV), respectively. The Nd 5d band dips sharply within a narrow window of *k*-space to become the CBM, which this is seen in all DFT methods. The large resultant energy dispersion of the conduction band, varying from 1.0 to 2.0 eV at the SCAN + *U* level of theory and from 1.4 to 2.3 eV at the HSE06 level of theory, is consistent with the large difference between the indirect and direct gaps that were measured in experiment. Figure 7 further shows that the general shapes of the F and FE valence and conduction bands are similar, and they confirm that the smaller computed band gap in F (vs FE) comes from the differences induced in the splitting of the Nd 5d conduction band. Visualization of the electron density that is generated by states at the CBM and VBM with the HSE06 functional (Figure S4 in the Supporting Information) characterize the VBM as Eu 4f with contributions from Cu/Se and the CBM as Nd 5d contributions from 5d orbitals oriented along the shortest Nd···Nd contacts that lie parallel with the [010] axis.

## 4 Conclusions

In summary, we report on novel heterometallic quaternary selenides EuPrCuSe_3_ and EuNdCuSe_3_, which were synthesized from a stoichiometric mixture of the parent elements in the presence of CsI as a flux, yielding crystals suitable for single-crystal X-ray diffraction analysis. The obtained selenides are isostructural and of the orthorhombic space group *Pnma* with the structure type Eu_2_CuS_3_. A 3D crystal structure is constructed from the EuSe_7_ capped trigonal prisms, Pr/NdSe_6_ distorted octahedra as well as CuSe_4_ tetrahedra. The Pr/NdSe_6_ capped trigonal prisms form 2D layers, further strengthened by 1D polymeric chains (CuSe_4_)_*n*_, which are separated by 1D dimeric ribbons, formed by the EuSe_7_ capped trigonal prisms and 1D free channels. The obtained selenides are semiconductors with direct gaps of 1.92 eV (for Ln = Nd) or 1.97 eV (for Ln = Pr) and indirect gaps of 0.90 eV (for Ln = Nd) or 0.94 eV (for Ln = Pr). The temperature-dependent magnetic susceptibilities of EuPrCuSe_3_ and EuNdCuSe_3_ follow the Curie–Weiss law, with the Weiss temperature being very close to zero. Both compounds are paramagnetic, with the transition to a ferromagnetic state at about 2.5 K for EuPrCuSe_3_ and about 3 K for EuNdCuSe_3_. The experimental magnetic characteristics resemble those which were recently reported for the sulfide derivatives EuPrCuS_3_ and EuNdCuS_3_, and furthermore they are in agreement with the calculated electronic structures of various magnetic states for EuPrCuSe_3_ and EuNdCuSe_3_. Both structures exhibit semiconductor behavior at the DFT level of theory, wherein the band gap separates the Eu 4f band from a very disperse Nd/Pr 5d conduction band, whose minimum corresponds with an in-plane interaction between Nd/Pr 5d orbitals along the shortest Nd···Nd (Pr···Pr) contacts. The ferromagnetic coupling is shown to relate with Se-mediated superexchange processes between lanthanide ions, which manifests in the model electronic structures as a maximization of spin density within the Se sublattice and a splitting of the Se-related bands.

## Supporting Information Available

Photograph of a single crystal of EuNdCuSe_3_ placed in a capillary for the X-ray diffraction analysis; experimental, calculated, and difference powder X-ray diffraction patterns of EuPrCuSe_3_ and EuNdCuSe_3_; computed electronic band structures for the F state of (EuNdCuSe_3_)_4_ with the experimental 298 K cell parameters at the PBE + D3 + *U*, MBJ + *U* and SCAN + *U* levels of theory; computed partial densities of states for the Nd f and Nd d contributions to the total DOS of the F state of (EuNdCuSe_3_)_4_ with the experimental 298 K cell parameters at the HSE06 level of theory; expanded collection of computed spin-state energetics and band gaps for EuNdCuSe_3_; fractional atomic coordinates, Wyckoff positions, and anisotropic displacement parameters (Å^2^) of EuPrCuSe_3_ and EuNdCuSe_3_; bond angles in the crystal structures of EuPrCuSe_3_ and EuNdCuSe_3_; energetics of other computed states of (EuNdCuSe_3_)_4_ and (EuPrCuSe_3_)_4_ at the DFT/PBE + D3 level; computed spin-state energetics, band gaps, and spin-densities of (EuNdCuSe_3_)_4_ with different types of DFT methods (PDF)
