# Supplementary material for: Experimental and Theoretical Insights on the Structural, Electronic, and Magnetic Properties of the Quaternary Selenides EuPrCuSe3 and EuNdCuSe3
Source: Inorg Chem. 2024 May 6;63(20):9040–9. doi: 10.1021/acs.inorgchem.3c04560 (PMC11110001; doi:10.1021/acs.inorgchem.3c04560)
Supplement: Supplementary file 1 — ic3c04560_si_001.pdf [file ic3c04560_si_001.pdf]

Supporting Information

Experimental and theoretical insights on the structural, electronic and magnetic properties of the quaternary selenides $EuPrCuSe_3$ and $EuNdCuSe_3$

Maxim V. Grigoriev[a], Anna V. Ruseikina[a,*], Alexander A. Garmonov[b], Ralf J. C. Locke[c], Filip Sagan[d], James Hooper[d,*], Mariusz P. Mitoraj[d], Thomas Schleid[c], Damir A. Safin[e,f,*]

[a] *Laboratory of Theory and Optimization of Chemical and Technological Processes, University of Tyumen, Volodarskogo Str. 6, 625003 Tyumen, Russian Federation*

[b] *Institute of Physics and Technology, University of Tyumen, Volodarskogo Str. 6, 625003 Tyumen, Russian Federation*

[c] *Institute of Inorganic Chemistry, University of Stuttgart, D-70569 Stuttgart, Germany*

[d] *Faculty of Chemistry, Jagiellonian University, ul. Gronostajowa 2, 30-387 Krakow, Poland*

[e] *Scientific and Educational and Innovation Center for Chemical and Pharmaceutical Technologies, Ural Federal University named after the First President of Russia B.N. Yeltsin, Mira Str. 19, 620002 Ekaterinburg, Russian Federation*

[f] *University of Tyumen, Volodarskogo Str. 6, 625003 Tyumen, Russian Federation*

* Corresponding author.
E-mail address: a.v.rusejkina@utmn.ru (A.V. Ruseikina), james.hooper@uj.edu.pl (J. Hooper), damir.a.safin@gmail.com (D.A. Safin)

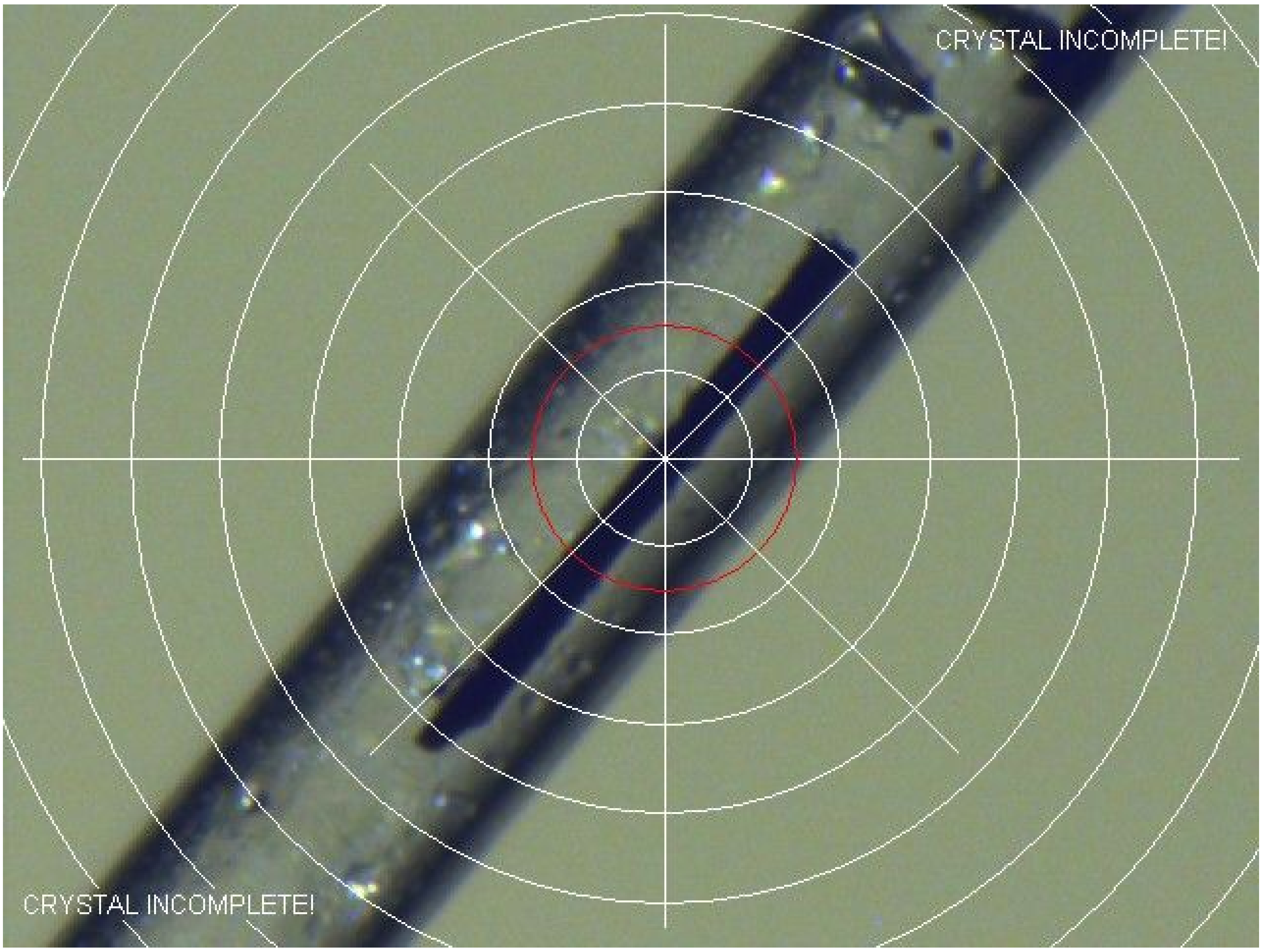

Figure S1. Photograph of a single crystal of $EuNdCuSe_3$ placed in a capillary for the X-ray diffraction analysis. Single crystals of $EuPrCuSe_3$ are similar.

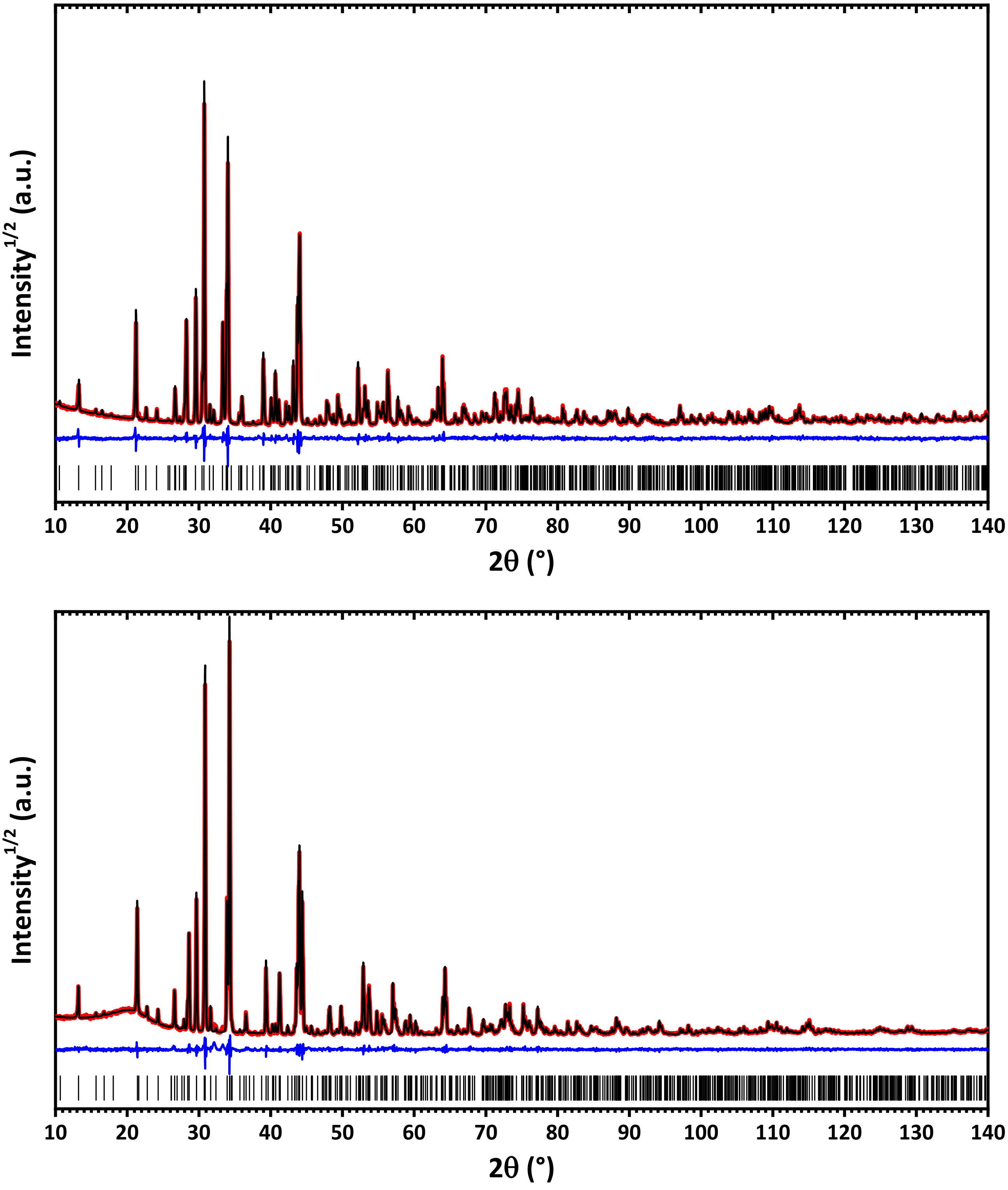

Figure S2. Experimental (red), calculated (black) and difference (blue) powder X-ray diffraction patterns of $EuPrCuSe_3$ (top; R_{wp} = 4.79, R_p = 3.71, R_B = 1.38) and $EuNdCuSe_3$ (bottom; R_{wp} = 5.06, R_p = 3.73, R_B = 1.38).

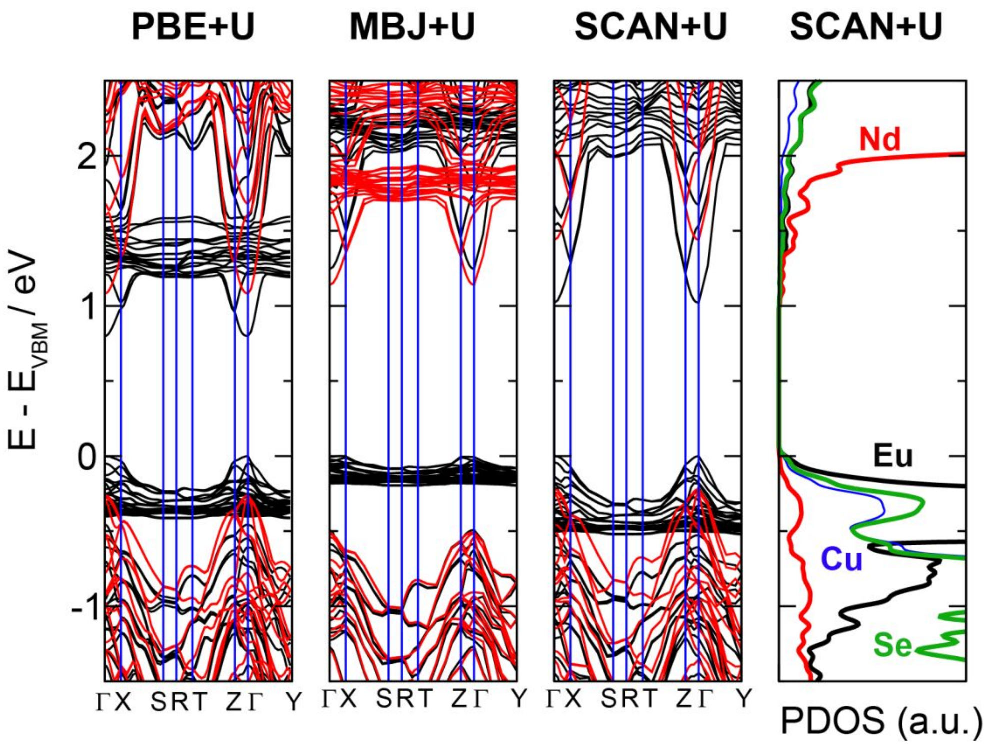

Figure S3. The computed electronic band structure for the *F* state of $(EuNdCuSe_3)_4$ with the experimental 298 K cell parameters at the PBE + D3 + U, MBJ + U/PBE + D3 + U and SCAN + U/PBE + D3 + U levels of theory. The valence band maximum (VBM) is set to zero in all of the plots, the two sets of spin orbitals are (separately) colored black and red. Also shown (at the far right) is the site-projected (partial) density of states at the SCAN + U/PBE + D3 + U level of theory.

Figure S3 shows the computed band structure of the *F* state for $(EuNdCuSe_3)_4$ at various levels of DFT theory. All methods place the Eu 4f bands just below the Fermi energy and the unoccupied Nd 4f/5d bands are visible above. This is corroborated by the site-projected (partial) density of states plot that is shown to the right. An unoccupied Nd 5d band is seen to decrease sharply and become the conduction band minimum (seen in all the Figure S3 plots near the Γ-point in this representation of k-space). We note that the metaGGA functionals (MBJ + U vs. SCAN + U) do disagree about the breadth of the valence band, the gap between the highest occupied Eu 4f and Se/Cu states, and of the natures of the lowest unoccupied Nd d/f bands. The considered hybrid functionals (B3LYP and HSE06) both appear to better resemble the SCAN + U electronic structure, but we prefer to reserve proper assessment of which method is more accurate to a future study. Here, we focus on the agreement between methods about what the major atom-centered contributions are to the VBM/CBM regions of the band structure.

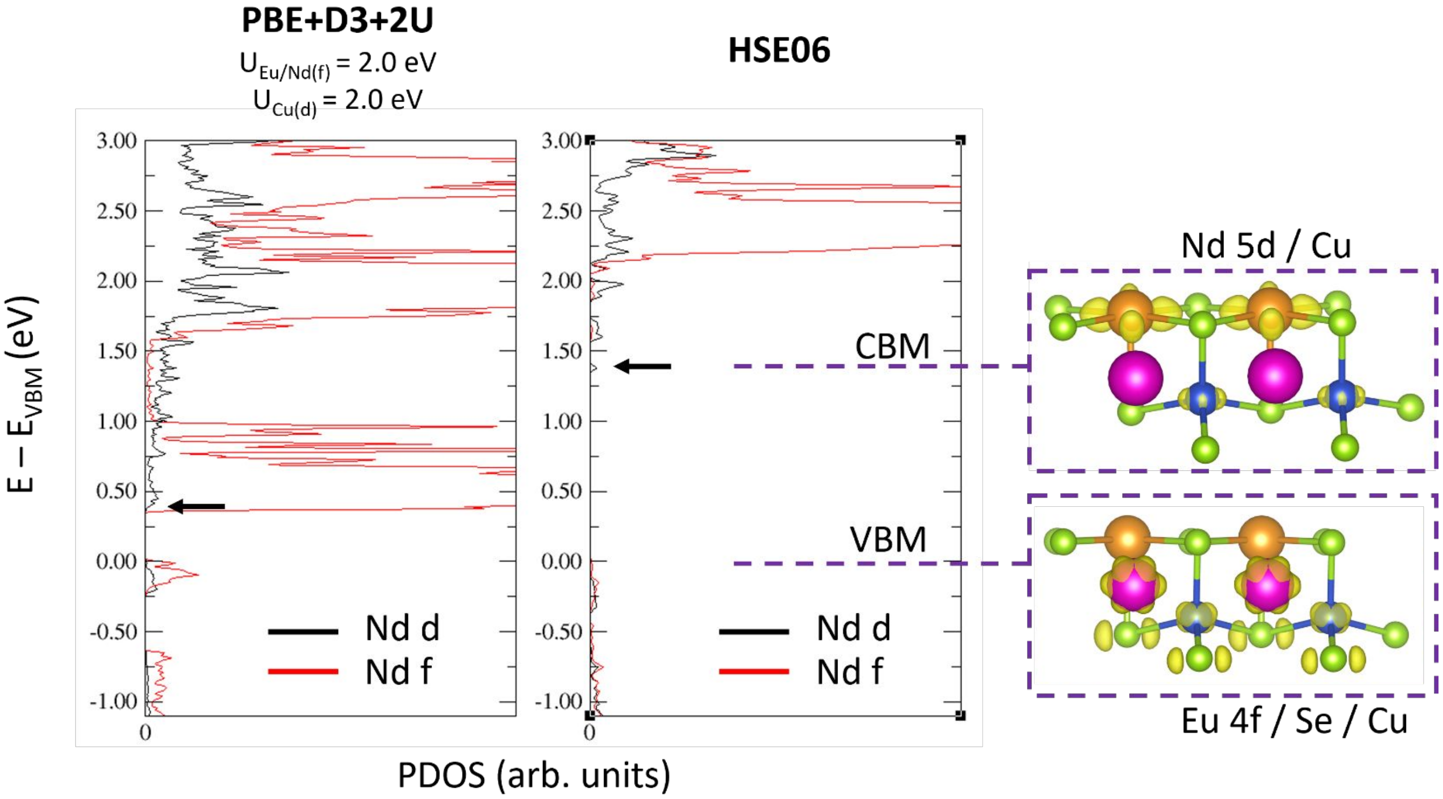

Figure S4. The computed partial densities of states (PDOS's) for the Nd f and Nd d contributions to the total DOS of the *F* state of $(EuNdCuSe_3)_4$ with the experimental 298 K cell parameters at the PBE + D3 + 2U level of theory (left) and at the HSE06 level of theory (middle). The valence band maximum (VBM) is set to zero in the PDOS plots and PBE + D3 + 2U is similar to what is used in the main text, but the Hubbard potentials for the Eu/Nd f states are assigned U = 2.0 eV instead of U = 4.0 eV. The arrows show the lowest-energy contribution of the Nd 5d states to the conduction band. The electron densities generated from the conduction band minimum (CBM) and valence band maximum (VBM) are shown for the HSE06 model with the isosurface value set to 0.003 e/Å^3(right). Color code: Eu = magenta, Nd = orange, Cu = blue, Se = green.

Figure S4 shows how the contribution of the lowest unoccupied Nd f states are shifted much lower in energy relative to the VBM as the strengths of the Eu/Nd 4f Hubbard potentials are decreased to 2.0 eV. The HSE06 PDOS plot, on the other hand, confirms that the Nd d states contribute to the conduction band minimum (CBM) and the bulk of the Nd f states lie > 0.5 eV above it, in agreement with the SCAN + U band structure in the main text. The electron densities generated from the VBM and CBM at the HSE06 level of theory are shown and, as mentioned in the main text, confirm that the CBM corresponds with an interaction between Nd lone-pair d orbitals within the planar $NdSe_2$ chain motifs that run parallel to the [0 1 0] axis.

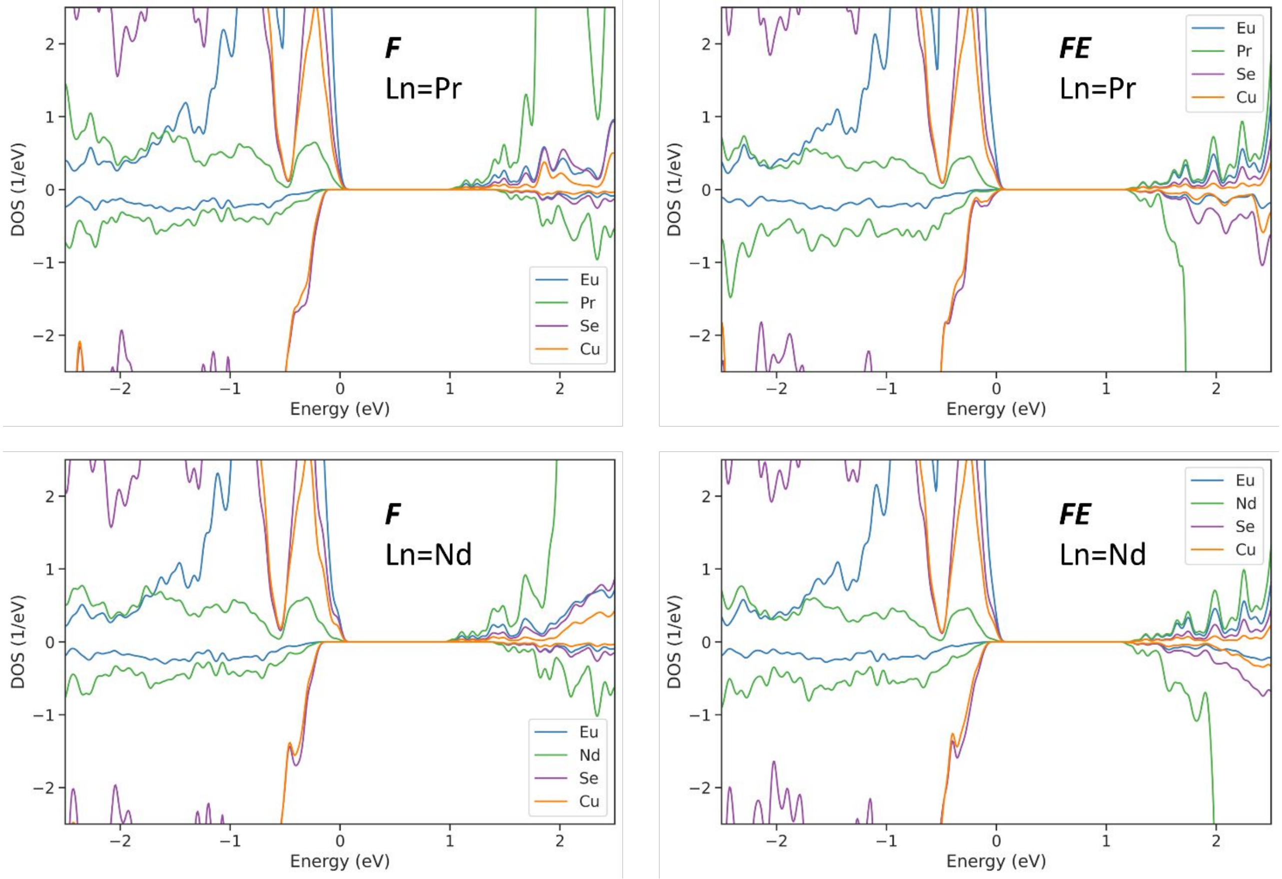

Figure S5. The computed site-projected (partial) densities of states (PDOS's) for the *F* (left) and *FE* (right) states of $(EuPrCuSe_3)_4$ (top) and $(EuNdCuSe_3)_4$ (bottom) at the DFT/SCAN + U level of theory. The alpha-spin electronic states are assigned positive values, the beta-spin states are assigned negative values, and the valence band maximum is set to zero in the PDOS plots.

Table S1. Fractional atomic coordinates, Wyckoff positions (W. p.) and anisotropic displacement parameters (Å^2) of $EuPrCuSe_3$ and $EuNdCuSe_3$.

Atom	*x*	*y*	*z*	W. p.	U_{11}	U_{22}	U_{33}	U_{12}	U_{13}	U_{23}
					$EuPrCuSe_3$					
Eu	0.28267(6)	1/4	0.50033(5)	4c	0.0249(4)	0.0154(3)	0.0192(4)	0	–0.0001(3)	0
Pr	0.01680(6)	1/4	0.26326(5)	4c	0.0149(4)	0.0133(3)	0.0191(4)	0	–0.0006(3)	0
Cu	0.23458(14)	1/4	0.77831(12)	4c	0.0200(9)	0.0255(8)	0.0255(9)	0	–0.0004(7)	0
Se1	0.05002(11)	1/4	0.88528(9)	4c	0.0173(7)	0.0155(6)	0.0187(6)	0	0.0007(5)	0
Se2	0.40223(11)	1/4	0.90096(9)	4c	0.0208(7)	0.0162(6)	0.0172(6)	0	0.0003(5)	0
Se3	0.26041(11)	1/4	0.17019(10)	4c	0.0173(7)	0.0156(6)	0.0182(7)	0	–0.0006(5)	0
					$EuNdCuSe_3$					
Eu	0.28076(5)	1/4	0.50033(5)	4c	0.0254(3)	0.0154(3)	0.0199(3)	0	0.0003(2)	0
Nd	0.01677(5)	1/4	0.26326(5)	4c	0.0166(3)	0.0137(3)	0.0194(3)	0	-0.0006(2)	0
Cu	0.23526(11)	1/4	0.77831(12)	4c	0.0224(7)	0.0249(7)	0.0241(7)	0	0.0005(5)	0
Se1	0.05035(9)	1/4	0.88528(9)	4c	0.0184(5)	0.0153(5)	0.0175(5)	0	0.0007(4)	0
Se2	0.40418(9)	1/4	0.90096(9)	4c	0.0206(5)	0.0157(5)	0.0179(5)	0	0.0011(4)	0
Se3	0.26101(8)	1/4	0.17019(10)	4c	0.0176(5)	0.0157(5)	0.0180(5)	0	-0.0004(4)	0

Table S2. Bond angles (°) in the crystal structures of $EuPrCuSe_3$ and $EuNdCuSe_3$. For symmetry codes see Figure 1.

**$EuPrCuSe_3$**	Se1–Eu–Se1^{i}	81.88	Se1^{ii}–Eu–Se3^{iv}	78.19	Se1^{vi}–Pr–Se3	86.50
	Se1–Eu–Se1^{ii}	73.35	Se2–Eu–Se2^{i}	81.20	Se1^{vi}–Pr–Se3^{vi}	88.17
	Se1–Eu–Se2	74.79	Se2–Eu–Se3^{iii}	76.99	Se2–Pr–Se2^{i}	90.82
	Se1–Eu–Se2^{i}	126.19	Se2–Eu–Se3^{iv}	129.94	Se2–Pr–Se3	89.57
	Se1–Eu–Se3^{iii}	90.35	Se2^{i}–Eu–Se3^{iii}	129.94	Se2–Pr–Se3^{vi}	95.65
	Se1–Eu–Se3^{iv}	151.54	Se2^{i}–Eu–Se3^{iv}	76.99	Se2^{i}–Pr–Se3	89.57
	Se1^{i}–Eu–Se^{ii}	73.35	Se3^{iii}–Eu–Se3^{iv}	83.54	Se2^{i}–Pr–Se3^{vi}	95.65
	Se1^{i}–Eu–Se2	126.19	Se1^{v}–Pr–Se1^{vi}	88.78	Se3–Pr–Se3^{vi}	172.54
	Se1^{i}–Eu–Se2^{i}	74.79	Se1^{v}–Pr–Se2	90.07	Se1–Cu–Se2	102.79
	Se1^{i}–Eu–Se3^{iii}	151.54	Se1^{v}–Pr–Se2^{i}	175.97	Se1–Cu–Se3	110.40
	Se1^{i}–Eu–Se3^{iv}	90.35	Se1^{v}–Pr–Se3	86.50	Se1–Cu–Se3^{vii}	110.40
	Se1^{ii}–Eu–Se2	139.07	Se1^{v}–Pr–Se3^{vi}	88.17	Se2–Cu–Se3	111.44
	Se1^{ii}–Eu–Se2^{i}	139.07	Se1^{vi}–Pr–Se2	175.97	Se2–Cu–Se3^{vii}	111.44
	Se1^{ii}–Eu–Se3^{iii}	78.19	Se1^{vi}–Pr–Se2^{i}	90.07	Se3–Cu–Se3^{vii}	110.17
**$EuNdCuSe_3$**	Se1–Eu–Se1^{i}	81.65	Se1^{ii}–Eu–Se3^{iv}	77.93	Se1^{vi}–Nd–Se3	86.79
	Se1–Eu–Se1^{ii}	72.08	Se2–Eu–Se2^{i}	81.06	Se1^{vi}–Nd–Se3^{vi}	88.39
	Se1–Eu–Se2	74.82	Se2–Eu–Se3^{iii}	77.53	Se2–Nd–Se2^{i}	90.93
	Se1–Eu–Se2^{i}	126.00	Se2–Eu–Se3^{iv}	130.45	Se2–Nd–Se3	89.89
	Se1–Eu–Se3^{iii}	90.27	Se2^{i}–Eu–Se3^{iii}	130.45	Se2–Nd–Se3^{vi}	94.85
	Se1–Eu–Se3^{iv}	151.01	Se2^{i}–Eu–Se3^{iv}	77.53	Se2^{i}–Nd–Se3	89.89
	Se1^{i}–Eu–Se^{ii}	73.08	Se3^{iii}–Eu–Se3^{iv}	83.43	Se2^{i}–Nd–Se3^{vi}	94.85
	Se1^{i}–Eu–Se2	126.00	Se1^{v}–Nd–Se1^{vi}	88.98	Se3–Nd–Se3^{vi}	173.24
	Se1^{i}–Eu–Se2^{i}	74.82	Se1^{v}–Nd–Se2	89.95	Se1–Cu–Se2	102.87
	Se1^{i}–Eu–Se3^{iii}	151.01	Se1^{v}–Nd–Se2^{i}	176.56	Se1–Cu–Se3	110.24
	Se1^{i}–Eu–Se3^{iv}	90.27	Se1^{v}–Nd–Se3	86.79	Se1–Cu–Se3^{vii}	110.24
	Se1^{ii}–Eu–Se2	139.09	Se1^{v}–Nd–Se3^{vi}	88.39	Se2–Cu–Se3	111.58
	Se1^{ii}–Eu–Se2^{i}	139.09	Se1^{vi}–Nd–Se2	176.56	Se2–Cu–Se3^{vii}	111.58
	Se1^{ii}–Eu–Se3^{iii}	77.93	Se1^{vi}–Nd–Se2^{i}	89.95	Se3–Cu–Se3^{vii}	110.13

Table S3. Energetics of other computed states of $(EuNdCuSe_3)_4$ and $(EuPrCuSe_3)_4$ at the DFT/PBE + D3 level. ΔE is defined as the difference between the total DFT energy of the indicated state (either *FE* or *C*) and the total DFT energy of state *F*.

System	Simulation cell	Electronic structure[a]	ΔE per unit cell (eV)
$(EuNdCuSe_3)_4$	1 × 1 × 1	*FE*	0.241
$(EuPrCuSe_3)_4$	1 × 1 × 1	*FE*	0.262
$(EuNdCuSe_3)_4$	1 × 2 × 1	*FE*	0.238
$(EuPrCuSe_3)_4$	1 × 2 × 1	*C*	0.110

[a]*FE* refers to the ferrimagnetic magnetic state, while *C* refers to a state where alternating alpha and beta spin densities are assigned to Eu/Nd atoms along the *b* cell axis.

Table S3 shows that the *F* state remains more stable at the PBE + D3 level theory also when an antiferromagnetic state (*C*) is built in a 1 × 2 × 1 supercell such that it allows antiferromagnetic coupling along the shortest Eu···Eu and Nd···Nd contacts. It is also seen here that the energy difference between *F* and *FE* is similar for $(EuPrCuSe_3)_4$, but it should be stressed that the models are all metallic or semimetallic with gaps of <0.2 eV at the DFT/PBE + D3 level of theory.

Table S4. The computed spin-state energetics, band gaps and spin-densities of $(EuNdCuSe_3)_4$ with different types of DFT methods. $E_F - E_{FE}$ shows the difference between the total DFT energy of the *F* and *FE* states, $[E_{CBM} - E_{VBM}]_F$ shows the computed band gap of the *F* state, and $|\rho_{spin}^{Se/Cu}|_F$ and $|\rho_{spin}^{Se/Cu}|_{FE}$ are measures of the spin density on the Se/Cu sublattice in the *F* and *FE* states. The "w/soc" tag indicates that spin-orbit coupling was included in the calculation.

Method	$E_F - E_{FE}$ (eV)	$[E_{CBM} - E_{VBM}]_F$ (eV)	$|\rho_{spin}^{Se/Cu}|_F$ (e/atom)	$|\rho_{spin}^{Se/Cu}|_{FE}$ (e/atom)
$(EuNdCuSe_3)_4$ with VASP 5.4 PAW potentials				
PBE + D3 + 2U	–0.16	0.41	0.037	0.008
PBE + D3 + 4U	–0.06	0.80	0.032	0.004
PBE + D3 + 4U w/soc[a]	–0.06	0.73	0.032	0.004
HSE06[a]	–0.09	1.37	0.024	0.003
HSE06 w/soc[a]	–	1.30	–	–
B3LYP[a]	–0.10	1.80	0.023	0.004
MBJ + 4U[a]		1.14	0.017	0.008
SCAN + 4U[a]	–0.01	1.02	0.041	0.003
SCAN + 4U w/soc[a]	–0.04	0.96	0.041	0.003
$(EuNdCuSe_3)_4$ with VASP 6.4 PAW potentials				
PBE + D3 + 4U	0.00	0.63	0.031	0.005
PBE + D3 + 4U w/soc[a]	–0.03	0.45	0.031	0.004
MBJ + 4U[a]	–	0.82	0.013	–
SCAN + 4U[a]	0.03	0.84	0.036	0.004

[a]these functionals were evaluated at the optimized PBE + D3 + 4U geometry.

An expanded collection of results about spin-state energetics, band gaps and Se/Cu spin densities of $(EuNdCuSe_3)_4$ is given in **Table S4**. Here the DFT + U methods that are described in the main text are referred to as DFT + 4U (i.e. wherein the Hubbard potentials on the Eu/Nd f states are assigned strengths of U = 4.0 eV) and they appear in Table S4 under the section that refers to the VASP 5.4 PAW potentials. The DFT + 2U methods refer to an assignment of U = 2.0 eV potentials to the Eu/Nd f states, and the VASP 6.4 PAW potentials refer to calculations which use PAW potentials taken from the recently released 6.4 versions of the VASP POTCAR files, specifically Eu_h ($5s^25p^66s^24f^7$ treated as valence), Nd_h ($5s^25p^66s^25d^15f^3$ treated as valence), Cu_pv ($3p^64s^23d^9$ treated as valence) and Se ($4s^24p^4$ treated as valence). The PAW potentials that were selected from the version 5.4

set were: Eu ($5s^25p^66s^24f^7$ treated as valence), Nd ($5s^25p^66s^25d^15f^3$ treated as valence), Cu ($4s^23d^9$ treated as valence) and Se ($4s^24p^4$ treated as valence). The VASP 6.4 PAW potentials were used with a plane-wave basis set cut-off of 500 eV and a 3 × 7 × 2 k-point mesh. The results confirm that the choice of PAW potential did not qualitatively affect the relative differences in band gaps and spin densities that were seen with the different DFT methodologies, but the spin state energetics appear more favorable of *FE* and the bands gaps are reduced relative to the results from the VASP 5.4 PAW potentials.

A related issue (which we refer to in the main text) is that of the problem with static correlation, especially with regards to the Nd and Pr energy levels. The data we present in the main text and in Table S4 was derived from using initial guesses of the system orbitals that were computed at the PBE+D3 level of theory, however we have observed that we can recover other electronic states of *F* and *FE* whose relative total energies and band gaps can change by upwards of 0.1 eV per cell (which is similar in scale to what we observed when testing the influence of the PAW potentials). We have checked that the band structure and spin density features that are discussed in the main text remain valid for the lowest-energy electronic states that we found, but we emphasize that the relative energies and computed band gaps of the F and FE spin states may change on the order of 0.1 eV.

The MBJ + 4U calculations were run from pre-converged PBE + 4U and SCAN + 4U sets of orbitals and with the default model parameters that are programed in VASP. The spin-orbit coupling was similarly run with the default routine that is used in the non-collinear implementation of magnetism in the VASP program, wherein the global spin-quantization axis was oriented along the z cartesian axis (i.e. with the SAXIS = 0 0 1 keyword). Testing was performed on how the results changed when SAXIS = 1 0 0 and SAXIS = 0 1 0 is used with the PBE + D3 + 4U and SCAN + 4U methods, and it was found that the resulting measures of magnetic anisotropy were less than 0.005 eV per cell and the band gaps were affected by less than 0.01 eV.
